# Incorporating Guided Autobiography, Portfolios, and SoulCollage Into Undergraduate Gerontology Courses

**DOI:** 10.1093/geroni/igaa057.2345

**Published:** 2020-12-16

**Authors:** Lisa Cox, Gina Maguire

**Affiliations:** Stockton University, Galloway, New Jersey, United States

## Abstract

Although undergraduates don’t eagerly choose gerontology classes first, a hefty number of students have enjoyed content they have explored as they were enrolled in “Aging and Spirituality” and “Therapeutic Arts with Older Adults” courses. The ten universal life themes that help people tell their stories, through the empirically validated methodology of Guided Autobiography (GAB) are incorporated in classes designed for an inter-generational group (older adults and students). Student assembled portfolios created from activities connected to ‘the arts’ and spiritual literacy have enhanced the acquisition of gerontological competencies. Also, through the use of SoulCollage® (developed in the late 1980s by Seena B. Frost, M. Div.) students of all ages, with and without artistic abilities, use a creative collaging process to get in touch with their ego integrity—the eighth stage of development conceptualized by Erik Erikson. Over three years, students have grown in their knowledge of aging and artistic processes.

